# Prevention and treatment of acute lung injury with time-controlled adaptive ventilation: physiologically informed modification of airway pressure release ventilation

**DOI:** 10.1186/s13613-019-0619-3

**Published:** 2020-01-06

**Authors:** Gary F. Nieman, Louis A. Gatto, Penny Andrews, Joshua Satalin, Luigi Camporota, Benjamin Daxon, Sarah J. Blair, Hassan Al-khalisy, Maria Madden, Michaela Kollisch-Singule, Hani Aiash, Nader M. Habashi

**Affiliations:** 1 Dept of Surgery, SUNY Upstate Medical University, 750 E Adams St, Syracuse, NY 13210 USA; 2 Multi-trauma Critical Care, R Adams Cowley Shock Trauma Center, University of Maryland Medical Center, 22 South Greene Street, Baltimore, MD USA; 3 Department of Critical Care, Guy’s and St, Thomas’ NHS Foundation Trust, Westminster Bridge Rd, London, SE1 7EH UK; 4 Dept of Anesthesiology and Perioperative Medicine, Mayo Clinic, 200 1st St SW, Rochester, MN 55905 USA; 5 Department of Clinical Perfusion, SUNY Upstate Medical University, 750 E Adams St, Syracuse, NY 13210 USA

## Abstract

Mortality in acute respiratory distress syndrome (ARDS) remains unacceptably high at approximately 39%. One of the only treatments is supportive: mechanical ventilation. However, improperly set mechanical ventilation can further increase the risk of death in patients with ARDS. Recent studies suggest that ventilation-induced lung injury (VILI) is caused by exaggerated regional lung strain, particularly in areas of alveolar instability subject to tidal recruitment/derecruitment and stress-multiplication. Thus, it is reasonable to expect that if a ventilation strategy can maintain stable lung inflation and homogeneity, regional dynamic strain would be reduced and VILI attenuated. A time-controlled adaptive ventilation (TCAV) method was developed to minimize dynamic alveolar strain by adjusting the delivered breath according to the mechanical characteristics of the lung. The goal of this review is to describe how the TCAV method impacts pathophysiology and protects lungs with, or at high risk of, acute lung injury. We present work from our group and others that identifies novel mechanisms of VILI in the alveolar microenvironment and demonstrates that the TCAV method can reduce VILI in translational animal ARDS models and mortality in surgical/trauma patients. Our TCAV method utilizes the airway pressure release ventilation (APRV) mode and is based on opening and collapsing time constants, which reflect the viscoelastic properties of the terminal airspaces. Time-controlled adaptive ventilation uses inspiratory and expiratory time to (1) gradually “nudge” alveoli and alveolar ducts open with an extended inspiratory duration and (2) prevent alveolar collapse using a brief (sub-second) expiratory duration that does not allow time for alveolar collapse. The new paradigm in TCAV is configuring each breath guided by the previous one, which achieves real-time titration of ventilator settings and minimizes instability induced tissue damage. This novel methodology changes the current approach to mechanical ventilation, from arbitrary to personalized and adaptive. The outcome of this approach is an open and stable lung with reduced regional strain and greater lung protection.

## Background

**Fig. 1:**
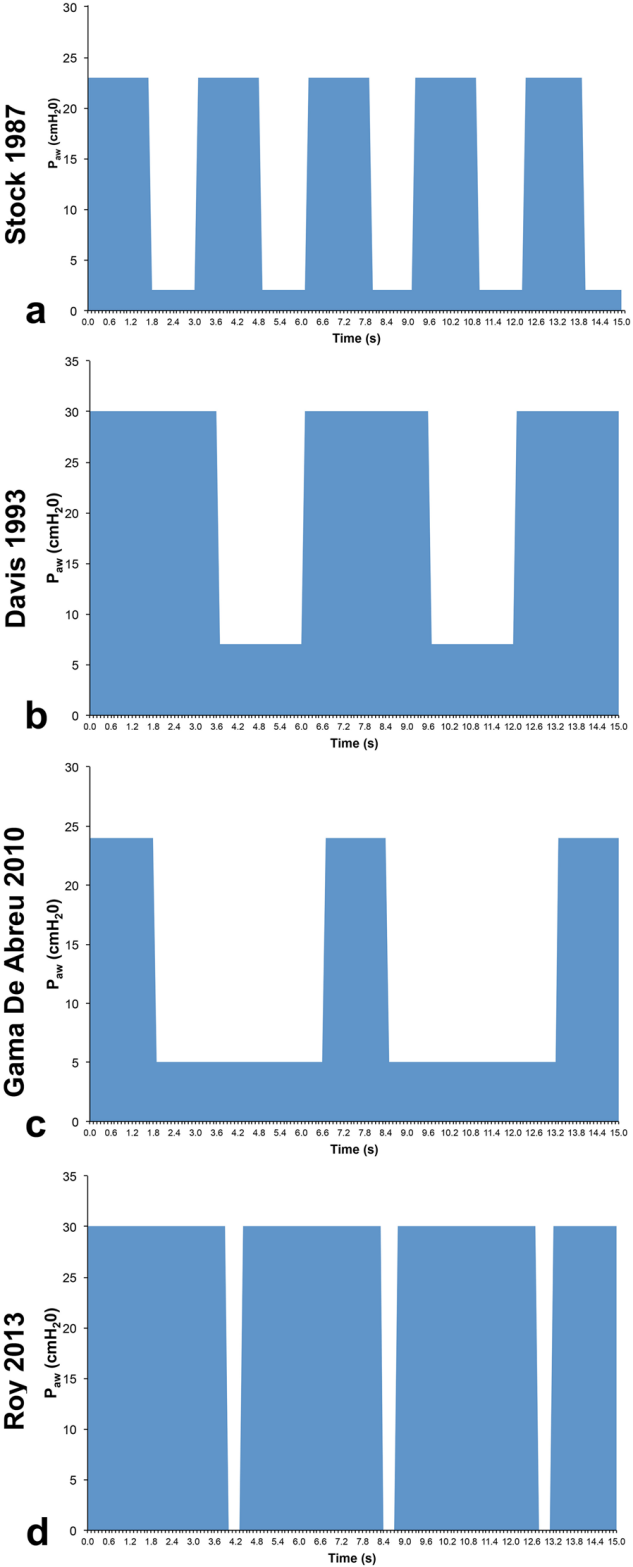
Airway pressure/time waveforms from published papers [12] all using the airway pressure release ventilation (APRV) mode but with different methods: **a** Stock et al. used a CPAP phase that encompassed 60% of each breath, a release phase of 1.27 s and a respiratory rate (RR) of 20/min [98]; **b** Davis et al. decreased the respiratory rate by prolonging both the CPAP and release phase [99]; **c** Gama de Abreau et al. adjusted their CPAP and release phase to values typical of a conventional breath [100]; **d** Roy et al. minimized the release phase and extended CPAP to occupy 90% of each breath, typical of the time-controlled adaptive ventilation (TCAV) method [83]. Although these studies all used the APRV mode, each differs significantly in the application methods used to set the mode

Globally more than three million patients per year develop acute respiratory distress syndrome (ARDS), accounting for 10% of all intensive care unit (ICU) admissions. In the United States, up to 200,000 patients a year are diagnosed with ARDS and 75,000 of these patients die [1]. Current ARDS treatment is supportive: protective mechanical ventilation, typically using lower tidal volume ventilation (Vt) and low–moderate positive end expiratory pressure (PEEP) [2]. Unfortunately, current protective ventilation strategies have not lessened ARDS mortality rate [1, 3–11]. The determinant of VILI is not the “mode” of ventilation, but the way parameters of the mechanical breath are set and combined. The goal of any protective mechanical breath should be maintaining functional residual capacity and increasing lung homogeneity. In this paper, we review the pathophysiology of ARDS in the microenvironment and identify how changes in alveolar micromechanics predispose the lung to a secondary VILI. Understanding how ARDS alters the dynamic alveolar inflation physiology enables us to adjust the mechanical breath profile (MB_P_—all airway pressures, volumes, flows, rates and the time at inspiration and expiration at which they are applied) necessary to minimize VILI [12]. Variants of the airway pressure release ventilation (APRV) mode have been used for decades with many combinations of settings (Fig. 1). In this review, we discuss the physiological impact of the time-controlled adaptive ventilation (TCAV) method on ARDS-induced abnormal alveolar mechanics, efficacy in both translational animal models and in a retrospective clinical analysis.

Acute respiratory distress syndrome pathophysiology current falls into three categories: (a) *normal* non-dependent tissue, (b) *severely injured and collapsed* dependent tissue, and (c) *unstable* tissue located between these two tissue types [13, 14]. Efforts to minimize VILI, block progressive acute lung injury (ALI), and reduce ARDS mortality have resulted in two current approaches: (1) *protect* and *rest* the lung or (2) *open the lung and keep it open* (open lung approach—OLA).

## Protect and rest strategy

The ARDSnet Low Vt (LVt) method is intended to protect the non-dependent normal lung tissue from overdistension (OD) and reduce alveolar recruitment/derecruitment (R/D) with positive end expiratory pressure (PEEP), while resting severely injured tissue by allowing it to remain collapsed throughout the ventilation cycle [2]. However, this strategy has not further reduced ARDS mortality [1, 3–11]. This suggests that our understanding of ARDS pathophysiology remains incomplete, particularly in the lung microenvironment [15, 16]. Indeed, the concept that the pulmonary parenchyma falls into three crudely differentiated categories according to the gravitational axis is being challenged. The current understanding is that open and collapsed tissues are not delineated into compartments, but are rather intermingled throughout the entire lung [17–21]. The unchanged mortality associated with the LVt method may also reflect the fact that maintaining lung tissue collapse (“resting”) may not be protective [1, 3–11]. The atelectatic lung does not exchange gas, is susceptible to pneumonia, and may ultimately lead to collapse induration and fibrosis with the inability to re-inflate or epithelialize the airspace [22–24].

## The open lung approach (OLA)

Using conventional ventilation strategies, the OLA has not been shown to reduce alveolar R/D-induced atelectrauma [25, 26] or improve survival [27]. In a recent RCT, the OLA with maximal recruitment strategy and PEEP set to best compliance resulted in increased mortality [27]. However, the lack of significant differences in compliance and driving pressure (∆*P*) between groups suggested that (1) the lungs had not been well recruited, which is essential for the OLA strategy to be effective; (2) the lungs were overdistended by excessive strain following the maximal recruitment; or (3) the chosen PEEP was not optimal to stabilize the newly recruited lung. Other research has shown [25] that OLA could not be attained using PEEP up to 15 cmH_2_O and plateau pressure (Pplat) limited to 30 cmH_2_O. While OLA is theoretically lung protective, traditional recruitment maneuver (RM) + PEEP methods may not provide sustained recruitment, stability, and homogeneity [25, 26, 28–30].

## New concepts of ARDS pathophysiology

More recent studies suggest that the lung pathology compartmentalized by gravity (i.e., normal lung tissue adjacent to acutely injured tissue) is incorrect and that regional lung strain and inflammation throughout the entire lung is the main driver of VILI [16, 31–36]. Regional strain is caused with each breath by (1) alveolar and alveolar duct R/D [37–43] and (2) stress-multiplication (S-M), which cause injury to open lung areas adjacent to collapsed or edema-filled tissue [18, 19, 44–48]. Retamal et al. used CT scans to generate volumetric strain maps revealing highly heterogeneous regional strains (caused by alveolar R/D and S-M), which suggests that there may not be a safe threshold for low Vt [49]. Cereda et al. hypothesized that VILI is not caused by overdistension of normal lungs, but rather develops in multiple areas of excessive regional strain located throughout the lung and caused by the primary insult [17]. They showed that tissue adjacent to the primary lesion was most susceptible to secondary VILI, an outcome supported by dynamic modeling of interdependent parenchyma during ALI [21]. This suggests that to effectively reduce VILI at the bedside, the clinician needs to know how to adjust ventilator settings (e.g., Vt, Pplat, PEEP, inspiratory and expiratory duration) to reduce R/D and S-M [50–52].

Synchrotron phase-contrast imaging can measure R/D at acinar length scales over short time frames and has demonstrated that lung collapse in the microenvironment differs between normal and acutely injured lungs [53–56]. Scaramuzzo et al. first measured tissue collapse in the microenvironment of the normal lung with graded reductions in PEEP. They assessed the numerosity (ASnum) and dimension (ASdim) of airspaces during lung deflation and found that the primary mechanism by which the lung loses volume was reduced ASnum secondary to alveolar and small airway derecruitment [53]. In a subsequent paper, Scaramuzzo showed in an ARDS model that the mechanism of lung deflation was reduced ASdim, which differs from the mechanism of normal lung deflation (ASnum) [54]. Broche et al. showed that “compliant collapse”, which is described “as a structural collapse of the airway wall along a certain length” is the primary mechanism of airway closure in the acutely injured lung [56]. “Compliant collapse” suggests that fluid movement in the microenvironment would play a role in airway collapse and reopening. Thus, the function of time during inspiration and expiration, and the opening and closing pressures, would be key components in keeping the lung open and stable [57].

This work underscores the merits of an extended inspiratory duration and a brief expiratory duration to improve alveolar recruitment and stability in a rat ARDS model [35], lung protection in a neonatal piglet model [58], and reduced ARDS incidence and mortality in trauma patients [59]. We postulate that as the lung opens, the increase in parenchymal tethering of airways [56] and alveolar interdependence [55] reduce lung pathology as a power-law function. Hamlington et al. have shown that progressive lung injury advances in power-law fashion where alveolar R/D (atelectrauma) caused the initial holes in the epithelium and that high airway pressure (volutrauma) greatly expands these holes in a power-law or rich-get-richer fashion [60]. Lung protection also arguably follows a power-law function with reestablishment of parenchymal tethering, alveolar interdependence, and surfactant function all working together to accelerate recruitment and stabilization of adjacent tissue.

## Understanding dynamic alveolar mechanics to design protective ventilation strategies

**Fig. 2:**
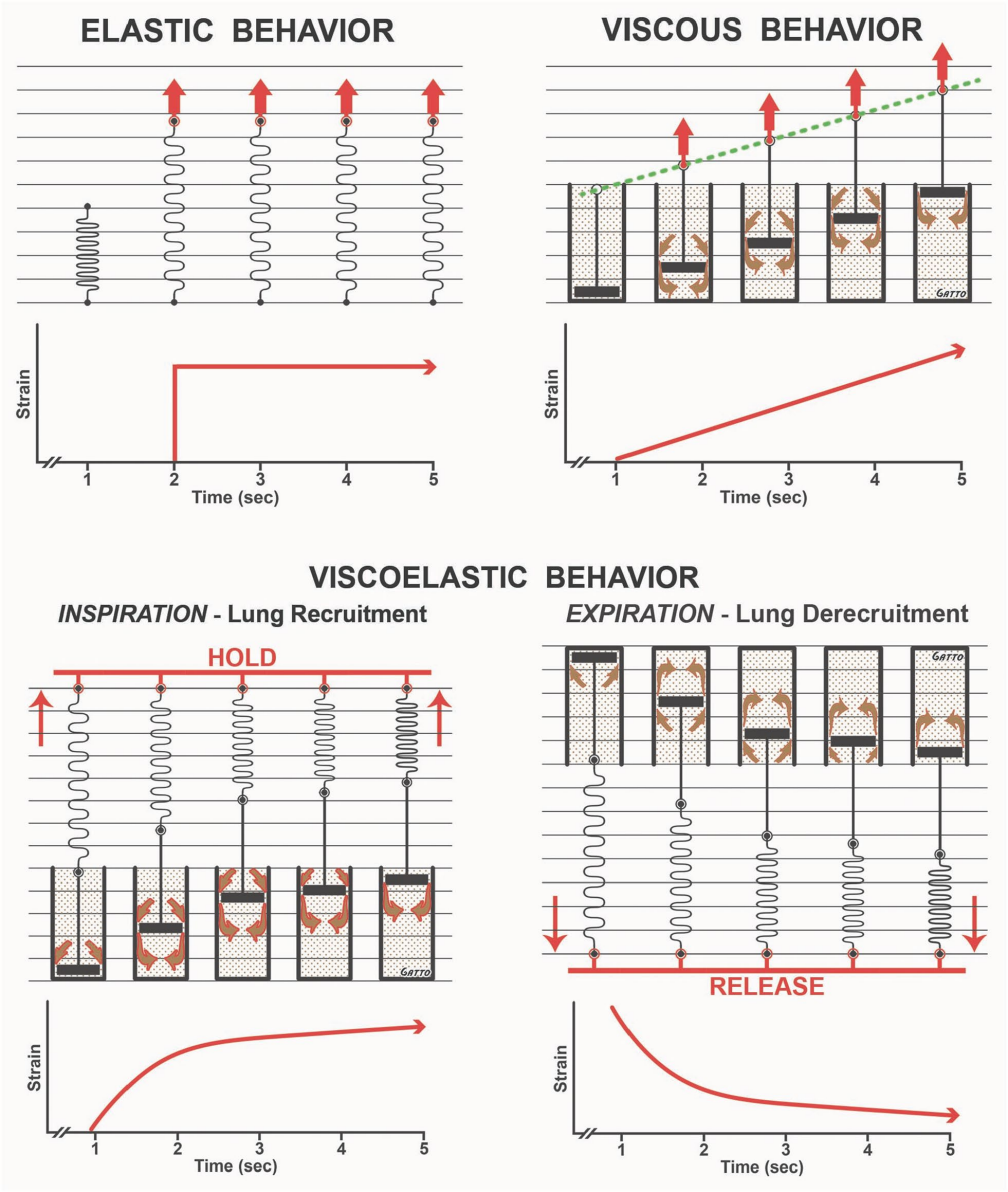
Strain/time curves for elastic (spring), viscous (dash in pot), and viscoelastic (spring and dashpot) systems. An applied force (red arrows) generates a stress that results in a yield or strain once the force reaches critical opening pressure. Upper left: the spring models elasticity with a rapid increase in strain leading to a plateau strain, which is distinctive of that spring. Upper right: the dashpot models viscous strain, where movement of the dash progresses (dashed line) with flow of the fluid in the pot around the dash (brown arrows), which is distinctive of the viscosity of the fluid. Bottom: viscoelastic behavior is modeled by the spring and dashpot, where force transfer from the spring to the dash results in a time-dependent strain with an initial rapid change in strain (1–2 s), which becomes gradual over time (2–5 s). Lung strain follows this behavior (Fig. 3). Bottom left: an extended inspiratory time (HOLD) optimizes lung recruitment once critical opening pressure is reached. Bottom right: a short expiratory time (RELEASE) minimizes lung derecruitment if it is sufficiently fast to prevent reaching the critical collapse pressure

Alveoli are often misunderstood as elastic and modeled as rubber balloons with immediate size changes (volumetric distortion or strain) with application or removal of pressure (physical stress) during inspiration and expiration [15]. In reality, alveoli behave in a viscoelastic rather than an elastic manner [21, 61–63]. Viscoelastic systems exhibit a time-dependent strain and can be conceptualized by the spring-and-dashpot model (Fig. 2) [21]. Figure 2 illustrates the strain/time curve of elastic (spring), viscous (dashpot), and viscoelastic (spring-and-dashpot) behaviors. Since the lung opens and collapses as a viscoelastic system, we use the spring and dashpot to illustrate lung recruitment during inspiration and derecruitment during expiration. The initial rapid opening or collapse (strain) of lung tissue followed by a continual opening or closing over an extended period time (2–5 s) is important.

Viscoelastic behavior of alveolar opening and collapse begins only after the critical opening or collapse pressure for that alveolus is reached. Before these critical pressures are obtained, there is no alveolar strain. However, the opening and closing pressures are not static; instead, they are dependent upon the level of surfactant deactivation and the degree of mechanical interdependence between adjacent alveolar walls and parenchymal tethering on the walls of small airways [55].

The original computational model of R/D by Ma and Bates was based on symmetrical bifurcations of the airway tree with each branch having an individual critical opening and collapse pressure [64]. However, this computational model no longer supported the new biological evidence on R/D at the acinar level. An alveolar interdependence component was added to the model such that the closure of a unit will impact the critical opening and collapse pressures of adjacent units [55]. Fluid movement in the microenvironment during airway collapse and reopening suggests that the pressures necessary for opening and collapse are also a function of the time at which they are applied [56]. Thus, a long inspiratory time with a short expiratory time would open more alveoli and prevent more alveolar collapse, as compared to the same airway pressures applied for shorter or longer amounts of time [57].

Since alveoli recruit as a viscoelastic system, once critical opening and collapse pressures are reached, the longer the inspiration (Fig. 2—red HOLD), the more lung tissue recruited with each breath [61, 65–68]. Similarly, the shorter the expiratory duration (red RELEASE), the less lung tissue that will collapse. Furthermore, the sustained inspiratory time causes both creep and stress relaxation, the most likely mechanism of which is redistribution of gas within the lung or opening of collapsed alveoli [69].

**Fig. 3:**
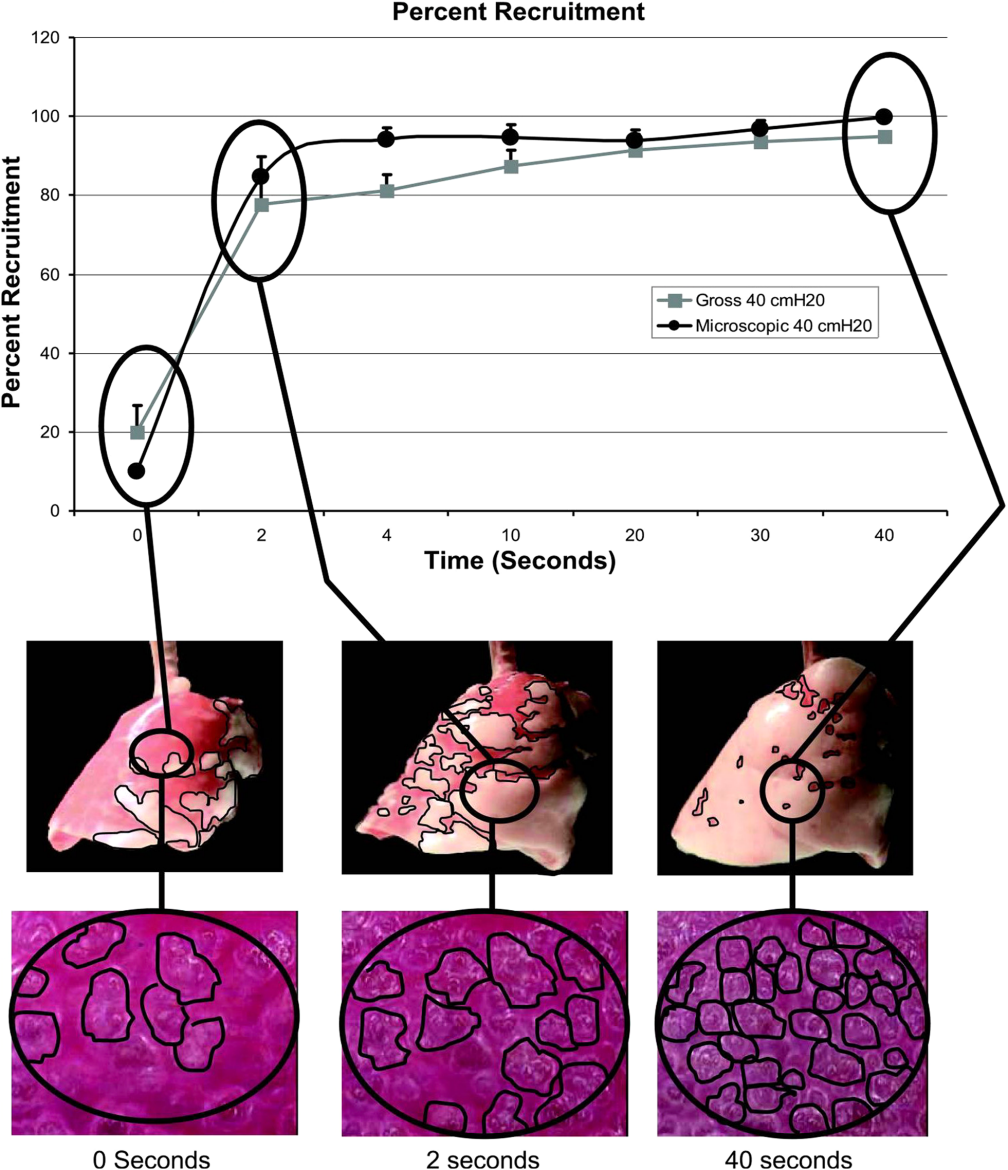
Recruitment over time (curve, top) in whole lung (squares, gross photos, middle) and subpleural alveoli (dots, photomicrographs, bottom) driven by 40 cmH_2_O airway pressure for 40 s [70]. Whole lung and alveoli continue to recruit over time while the pressure remains constant (viscoelastic behavior)

We postulate that this information can be used to design an MB_P_ that will open and stabilize the acutely injured lung. The longer the inspiratory time, the more alveoli recruited. We previously quantified in vivo alveolar recruitment in real-time in a rat ARDS study that involved mathematical modeling. Initial recruitment after the applied breath did not begin until after the first second, followed by a rapid recruitment (1–2 s). The majority of recruitment occurred in 2 s with continued gradual recruitment over the subsequent 38 s (Fig. 3) [70]. The absence of any inflation for the first second has clinical significance since inspiratory time in most conventional ventilator settings is 0.5–1.0 s.

A brief inspiratory time confines ventilation to proximal conducting/convective airways rather than allowing the time-dependent gas distribution to reach and facilitate diffusion in the distal airspace [16]. Other investigators using CT scans combined with mathematical modeling also support this temporal lag in alveolar opening following an applied proximal airway pressure [71–73]. The similarities between alveolar percent recruitment/time (Fig. 3) coincide with the viscoelastic behavior strain/time curves (Fig. 2, inspiration—lung recruitment). Derecruitment of alveoli is also viscoelastic in nature (Fig. 2, expiration—lung derecruitment). The deflation strain/time curve suggests that a ventilator strategy with a brief expiratory duration (red RELEASE) would minimize lung collapse, placing ventilation on the more favorable expiratory portion of the pressure–volume curve [74].

## Designing a mechanical breath to fulfill the OLA

**Fig. 4:**
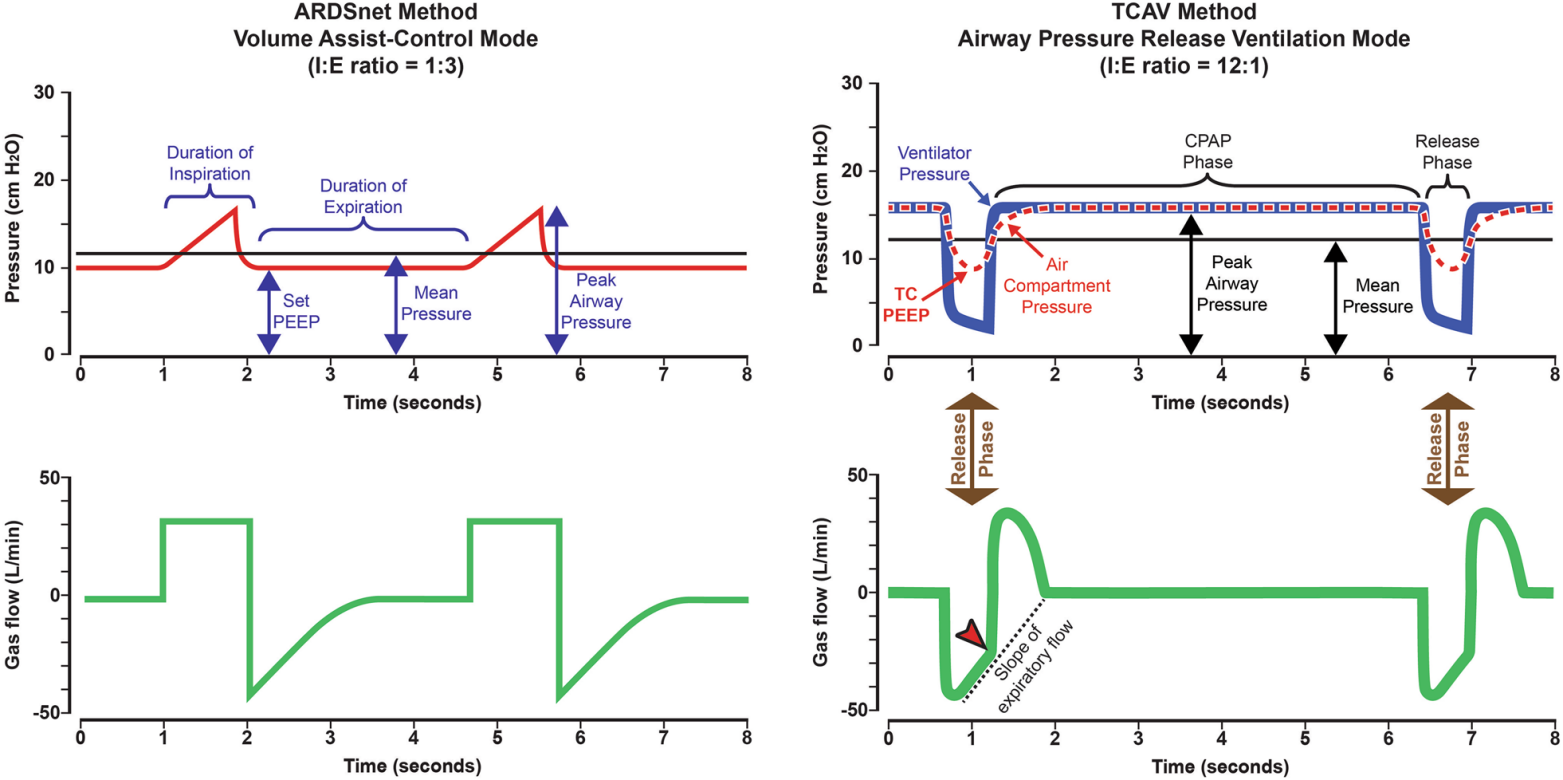
The ARDSnet method using the volume assist-control ventilation mode (left) has an I:E ratio of 1:3, which directs a short inspiration and a long expiration, and PEEP is arbitrarily set. Conversely, the TCAV method (right) has an I:E ratio of 12:1, which directs a long inspiration (CPAP phase) and a short expiration (release phase), not allowing the lung to fully depressurize and resulting in a time-controlled PEEP (TC-PEEP, red dashed line). Time controlled-PEEP (TC-PEEP) is adaptive (not arbitrary) because it is determined in real-time according to compliance, which is measured in the preceding breath by the slope of the expiratory flow curve (Slope_FE_) (red arrowhead on right) (Fig. 6)

There is no mechanistic evidence that current OLA protocols using a RM and titrated PEEP actually achieve and sustain an open lung [26, 75, 76]. The ARDSnet method features a brief time at peak inspiration and an extended time at expiration (Fig. 4, left), producing an MB_P_ that is antithetical to the TCAV method (Fig. 4, right). Conversely, the TCAV method reconfigures time allocation to extend inspiration using a continuous positive airway pressure phase (CPAP phase) with a brief (sub-second) release for exhalation (release phase). Open valve CPAP is used rather than closed valve to allow the patient to spontaneously inhale or exhale with little added resistance at any time in the breathing cycle. The short expiratory time does not allow the expiratory flow to reach zero flow, and therefore, the alveolar pressure is always above the set expiratory pressure (*P*_Low_), which itself is always set at 0 cmH_2_O. The CPAP phase initiates before the lung fully depressurizes (Fig. 4, right), maintaining a positive end expiratory pressure determined by the peak expiratory flow, the expiratory duration, and the compliance of the respiratory system. The gas volume released (Vr) during the release phase is analogous to Vt in that it equals the volume delivered during the CPAP phase (we use Vt in place of Vr in this review for consistency). However, TCAV does not aim to achieve a target Vt, but rather the Vt changes depending on the release time (*T*_Low_), which is adjusted by changes in respiratory system compliance (*C*_RS_): ↓*C*_RS_ = ↓Vt and ↑*C*_RS_ = ↑Vt

## Inspiratory time and lung recruitment

Alveolar recruitment is not only a function of the amount of pressure applied to the lung, but also of the time during which the pressure is applied because alveoli open and collapse as a viscoelastic system (Fig. 2, viscoelastic behavior). Alveolar volume change is further influenced by alveolar micro-anatomy, including parenchymal tethering and shared alveolar walls, establishing alveolar interdependence. All the above components play an important role in alveolar recruitment and derecruitment [19, 62, 77–79]. Thus, the longer airway pressure is applied, the more alveoli recruited (Fig. 2, viscoelastic behavior) [70]. This time-dependent recruitment has been described by Suki et al. as the “avalanche theory” of lung inflation [80].

## Personalized and adaptive lung recruitment

We conducted histological measurements of terminal airspace in a rat ARDS model [16] and reported a redistribution of gas from alveolar ducts into alveoli with TCAV, but not with a volume-controlled mode. Stress relaxation occurs during the CPAP phase because there is sufficient time for alveoli to be recruited. We postulate that gas is transferred from the more elastic ducts (Fig. 2, viscoelastic behavior—rapid initial strain) into the more viscous alveoli (Fig. 2, viscoelastic behavior—slow progressive strain over time) during the extended CPAP Phase.

By comparison, the ARDSnet brief inspiratory time (Fig. 4, left, duration of inspiration) method would not effectively recruit viscoelastic alveoli, allow time for tissue creep, or result in redistribution of gas from the ducts into the alveoli [16, 81]. This is supported by studies indicating that the OLA, which uses occasional RMs combined with a brief inspiratory duration (Fig. 4, left), has not been shown to reduce mortality. The likely reason for this lack of efficacy is that neither RMs nor the brief inspiratory duration effectively opens the lung; therefore, alveolar heterogeneity and regional strain were not eliminated [26, 82, 83]. To normalize the alveolar duct to alveolar volume distribution in the acutely injured lung, it is necessary to use a combination of an extended time at inspiration (CPAP phase) and short expiratory duration (Release phase) (Fig. 4, right).

**Fig. 5:**
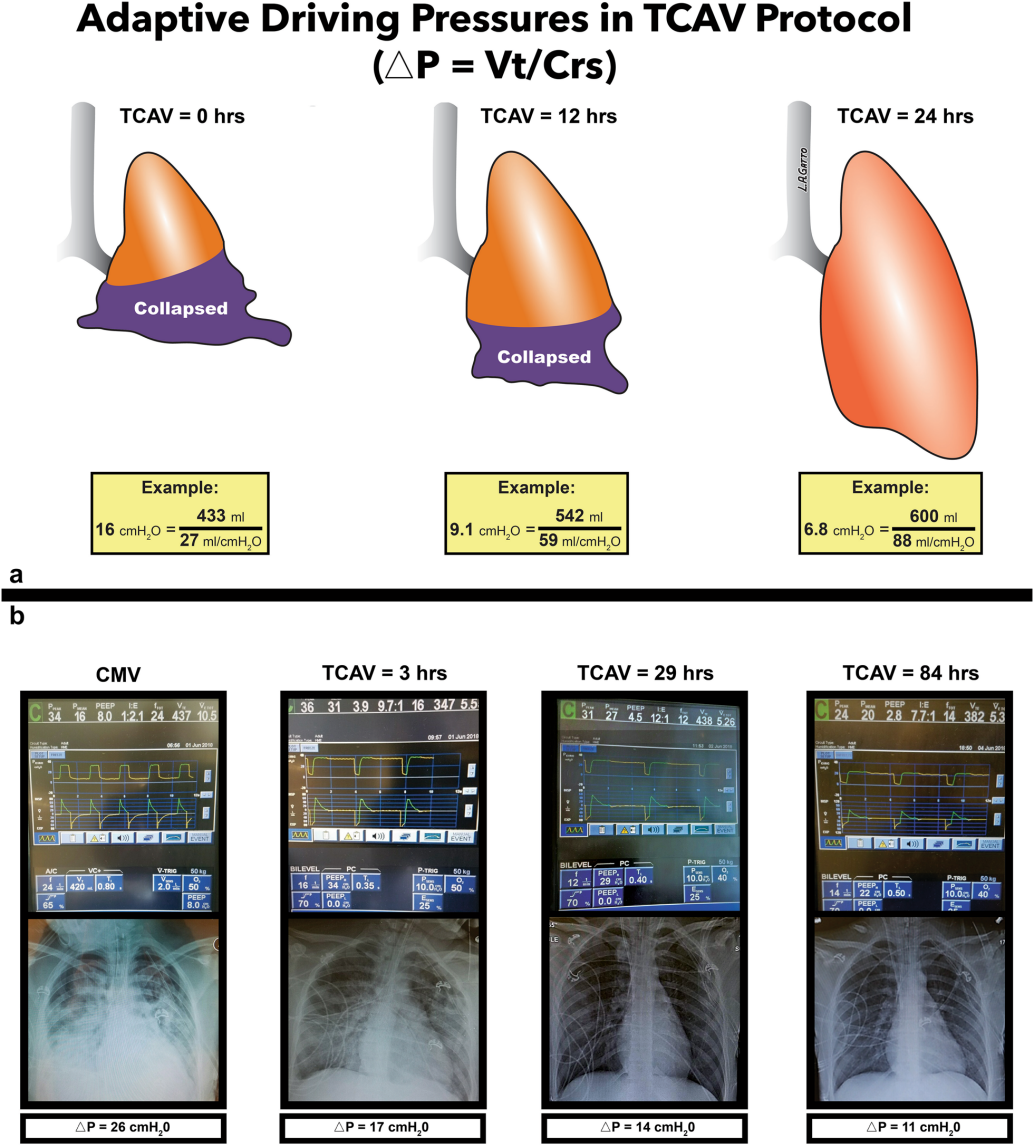
Optimizing recruitment with TCAV allows the lung to accommodate increased tidal volumes, without increases in driving pressure, due to a concomitant increase in compliance. **a** TCAV-induced lung recruitment over time (0–24 h) in a brain-dead organ donor. Driving pressure (Δ*P*) was calculated as tidal volume (Vt) divided by respiratory system compliance (*C*_RS_). The adaptive nature of TCAV delivers low Vt (7.3 ml/kg at 0 h) with lung collapse and low *C*_RS_, but adjusts Vt over time (Vt = 9.2 ml/kg at 12 h, Vt = 10.1 ml/kg at 24 h) as the lung opens and *C*_RS_ increases. Notably, Δ*P* actually decreased despite increasing Vt (**a**). **b** Evolution of driving pressure (Δ*P*) and chest X-ray (CXR) over time: **a** *CMV* (conventional mechanical ventilation) on a brain-dead organ donor (55 kg) with baseline ventilator settings: VC-AC, Vt 420, rate 24, PEEP 8 cmH_2_O with Peak pressure 34 cmH_2_O, Vt 7.9 mL/kg/predicted body weight (PBW), and Δ*P* 26 ml/cmH_2_O. Chest X-ray showed severe bilateral infiltrates. TCAV = 3 h: 3 h after transition to TCAV with settings: CPAP phase pressure = 34 cmH_2_O, release set pressure = 0 cmH_2_O, CPAP time = 3.4 s, release phase duration = 0.35 s. Note the lower Vt of 347 ml (6.3 ml/kg/PBW), which gradually increased from a Vt of 5.4 ml/kg/PBW when first transitioned to TCAV (data not shown); both Vts using the TCAV protocol are lower than those on the conventional mode (CMV = 437 ml, 7.9 ml/kg/PBW). The CXR demonstrates radiographic clearing of densities with significant recruitment and a reduction in Δ*P* from 26 to 17 ml/cmH_2_O. TCAV = 29 h: 29 h on TCAV, a new chest radiograph for line placement indicated continued recruitment, and the CPAP phase pressure was subsequently decreased to 29 cmH_2_O. In addition, the angle of the expiratory flow curve became less acute (Fig. 6), and the release phase duration was increased to 0.4 s. The CPAP time was increased to 4.6 s because ventilation had improved. Despite a lower *P*_High_, the Vt continued to increase as did an improvement in *C*_RS_. The continued radiographic clearing of densities and reduction in Δ*P* fell to 14 ml/cmH_2_O despite continued Vt increase. TCAV = 84 h: The CPAP phase pressure was further decreased to 22cmH_2_O due to continued recruitment (CXR) with a Δ*P* of 11 ml/cmH_2_O. The lungs and the heart, liver, and both kidneys from this organ donor were all successfully transplanted

The physiologic impact of TCAV on lung recruitment over time in a brain-dead organ donor is depicted in Fig. 5a, top. Displayed respiratory system compliance (*C*_RS_), driving pressure (∆*P* = Vt/*C*_RS_), and Vt measurements are after initial transition of the brain-dead donor to TCAV (TCAV = 0 h) and then 12 (TCAV = 12 h) and 24 (TCAV = 24 h) hours on TCAV. The prolonged inspiratory time (Fig. 4, right) gradually “nudges” open the lung and normalizes gas distribution within the alveoli and ducts (Fig. 5a—blue collapsed lung tissue converting to open tan tissue) and the brief expiratory time prevents these newly opened alveoli from re-collapsing (Fig. 4, right) [16].

Although the ∆*P* was slightly elevated (16.0 cmH_2_O) when TCAV was first applied (*T*0) due to the low *C*_RS_ (27 ml/cmH_2_O), it remained within the safe range due to the low Vt (7.3 ml/kg). As the lung recruited over time, the Vt increased (*T*12 = 9.2 ml/kg) without increasing ∆*P*, which fell into the normal lung range (9.1 cmH_2_O) due to increased *C*_RS_ (59 ml/kg). Continual reduction in ∆*P* occurred because *C*_RS_ increased (*T*24 = 88 ml/cmH_2_O) as the lung fully opened and ∆*P* fell into the normal range (6.8 cmH_2_O) (Fig. 5a, top) with a Vt of 10.1 ml/kg. These data indicate how the Vt can only increase if *C*_RS_ increases, which personalizes the Vt to the pathophysiology of the patient’s lung in real-time and normalizes the tidal volume to lung volume (Fig. 5a, top). Figure 5b, bottom depicts the ventilator screen and the chest radiograph (CXR) from a brain-dead donor initially on controlled mechanical ventilation (CMV) and then converted to TCAV. The progressive changes in ∆*P* and CXR at 3 (TCAV = 3 h), 29 (TCAV = 29 h) and 84 (TCAV = 84 h) hours on TCAV are displayed. The progressive decrease in ∆*P* as the lung recruits is identified by the reaeration of the lung on CXR. These data suggest that an extended CPAP duration for a period of hours will “nudge” alveoli open with each breath, reducing *C*_RS_ and allowing ventilation at a low ∆*P* even with a Vt higher than 6 ml/kg.

## Expiratory time and lung collapse

The lung becomes time and pressure dependent when acutely injured, such that it will quickly collapse at atmospheric pressure [67, 84–86]. In animal ARDS models, the majority of lung collapse occurred in the first 4 s of exhalation with collapse as fast as 0.6 s [72]. This suggests preventing collapse of alveoli with the fastest time constants, the expiratory duration must be less than 0.6 s. Markstaller et al. had similar findings in an ARDS porcine model with lung collapse occurring in 95% of the lung within 0.8 s [87]. Lachmann was one of the first to suggest that stabilizing alveoli with heterogeneous collapse time constants could be accomplished by dramatically shortening expiratory time [88]. Together, these studies suggest it is possible to stabilize alveoli with fast collapse time constants by using a brief expiratory time [72, 85, 87].

## Personalized and adaptive lung stabilization

**Fig. 6:**
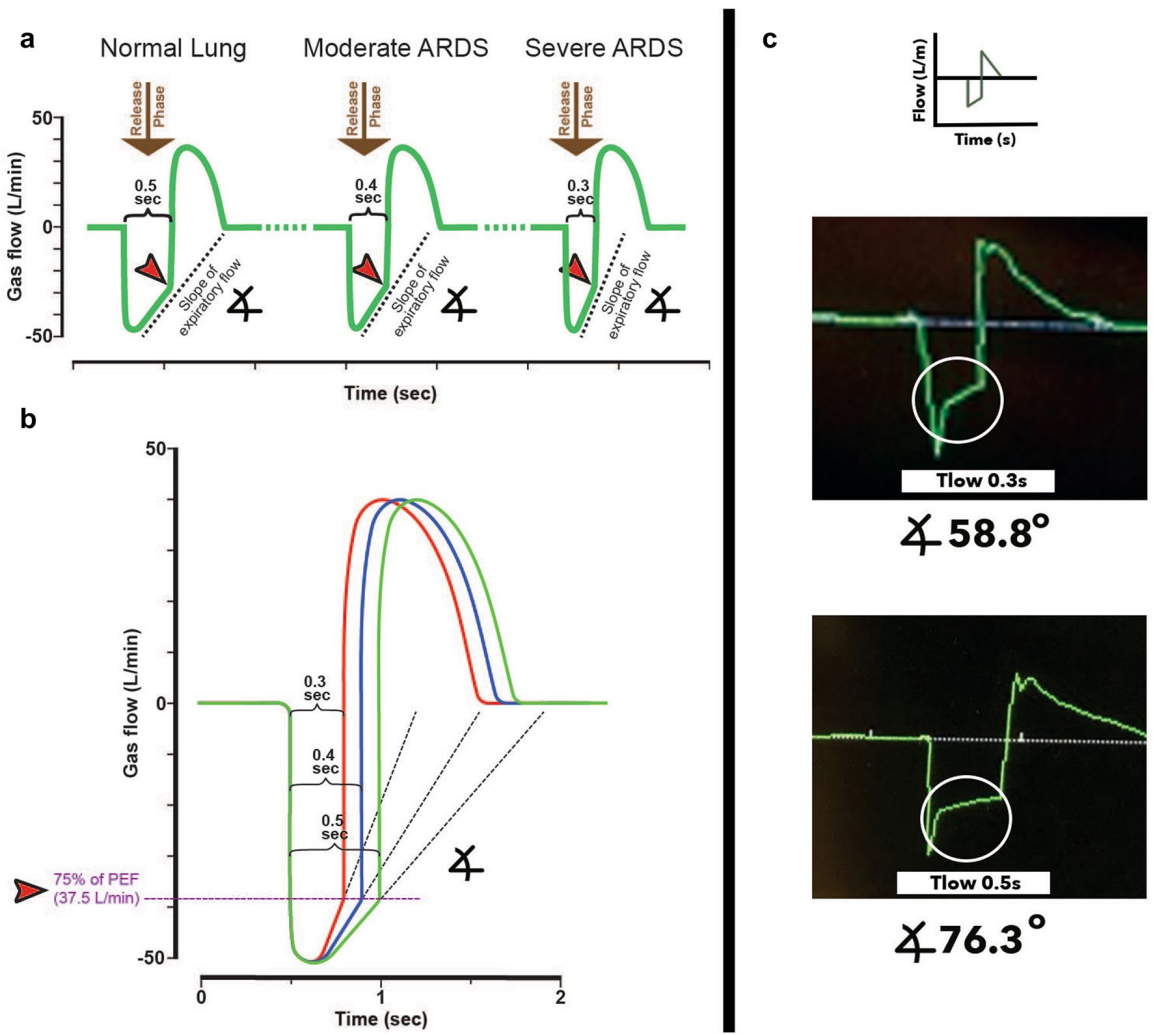
Personalizing the *release phase* using the *slope of the expiratory flow curve* (Slope_FE_). The release phase becomes briefer, directed by the Slope_FE_ with lung injury severity. **a** *Normal lung* release phase is 0.5 s, with *moderate ARDS* of 0.4 s and *Severe ARDS* of 0.3 s, all directed by changes in the Slope_FE_. **b** The release phase duration is calculated by expiratory flow terminating (*E*_FT_) at 75% of the expiratory flow peak (*E*_FP_) (red arrow head). In this example, the *E*_FP_ = − 50 l/min, so flow will be terminated (*E*_FT_) at − 37.5 l/min (− 50 l/min × 75% = − 37.5 l/min). Although the *E*_FT_ is always at 37.5 l/min in our example, the release phase duration varies (0.3, 0.4, 0.5 s) due to changes in the Slope_FE_ (**a**, **b**). We did not directly measure the slope of the expiratory flow curve, but by terminating expiration at 75% of the *E*_FT_, changes in the slope change the expiratory duration (**a**, **b**). Thus, the release phase is both *personalized* and *adaptive* as the patient’s lungs become better or worse using the TCAV method. **c** Expiratory flow/time graphics on a ventilator monitor from a brain-dead organ donor meeting Berlin criteria for severe ARDS. The release phase was set using the equation: *E*_FP_ × 75% = *E*_FT_. The Slope_FE_ when TCAV was initially applied was 58.8°, resulting in a release phase of 0.3 s. Twenty-four hours on TCAV and the Slope_FE_ increased to 76.3°, resulting in a release phase of 0.5 s. The spike in the expiratory flow curve is an artifact due to compression of gas in the ventilator circuit

The slope of the expiratory flow curve (Slope_FE_) allows breath-by-breath assessment of changes in *C*_RS_ (Fig. 6) [89]. With progressive ALI, edema and loss of surfactant function increases lung recoil force, causing rapid lung collapse and decreased *C*_RS_. The collapse rate of the lung is manifested as a change in the slope of the expiratory flow curve (Slope_FE_), a measure of lung recoil, which is determined by *C*_RS_ and both turbulent and viscous resistances [89]. Brody demonstrated that (1) lung *C*_RS_ could be calculated if both of these resistances are known; (2) dynamic *C*_RS_ must be a constant, independent of volume; and (3) the inertia of the chest–lung system is negligible [89]. The brief release phase is passive without muscular effort or added external resistance (i.e., PEEP) such that the Slope_FE_ can be used as a bedside monitor to analyze the mechanical properties of the respiratory system on a breath-to-breath basis [89].

The release phase is protocolized using the TCAV method for the expiratory flow to terminate (*E*_FT_) at 75% of the expiratory flow peak (*E*_FP_) (*E*_FP_ × 75% = *E*_FT_) (Fig. 6a, b) [90]. The formula *E*_FP_ × 75% = *E*_FT_ was first identified empirically at the bedside to be effective at stabilizing the lung [90] and has been subsequently shown to be most effective at maintaining open and stable alveoli [35], normalizing alveolar/alveolar duct volume distribution [16], and resulting in homogeneously ventilated alveoli [36]. In the example presented in Fig. 6b, *E*_FP_ is − 50 l/min, so the expiratory flow is terminated (*E*_FT_) at − 37.5 l/min (− 50 l/min × 75% = − 37.5 l/min). To accomplish this at the bedside, the clinician sets the ventilator to terminate the expiratory flow when it reaches 37.5 l/min (Fig. 4, right), and the CPAP phase is restored (Fig. 4, right). Although Slope_FE_ is not directly measured, variation in the slope causes a change in release phase duration: gradual slope = long release phase and steep slope = short release phase (Fig. 6a, b, 0.5, 0.4, 0.3 s release phase times with changes in the Slope_FE_).

Figure 6c depicts two airway flow/time curves with the Slope_FE_ circled and the angle measured on the ventilator monitor in a brain-dead donor. The top curve shows the initial application of TCAV, and the bottom curve is 24 h later. With a steep Slope_EF_, expressed as an angle (58.8°), the expiratory time is short (*T*_Low_ 0.3 s), and as the Slope_EF_ increases (angle goes from 58.8º to 76.3°), the expiratory duration increases (*T*_Low_ 0.5 s). This illustrates that the duration of the release phase changes with changing lung pathology and thus is *personalized* and *adaptive* as the patient’s lung mechanics becomes better or worse (Fig. 6a, b).

## Personalized and adaptive tidal volume

**Fig. 7:**
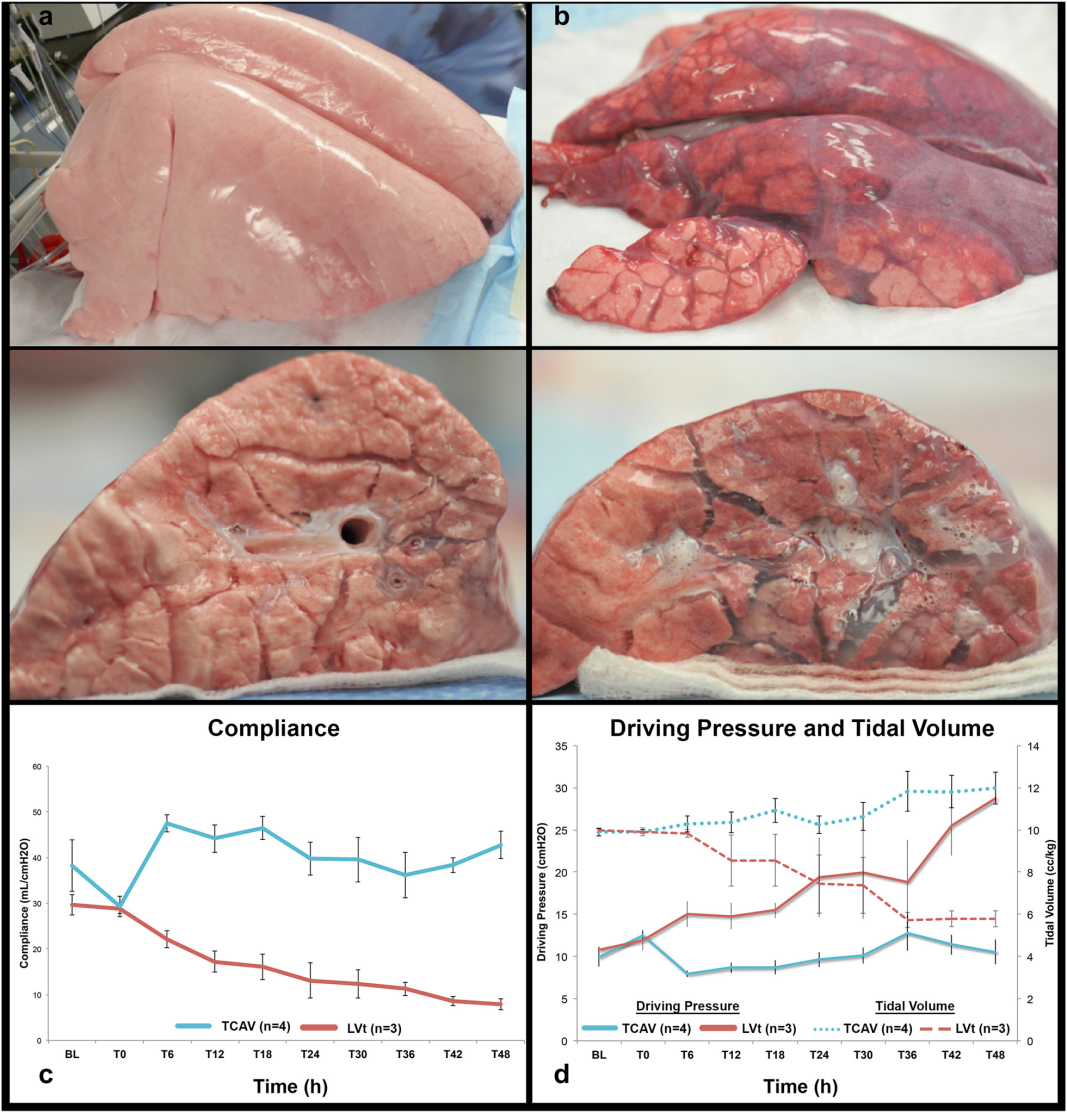
Gross lung photos with corresponding driving pressure (Δ*P*), tidal volume (Vt), and respiratory system compliance (*C*_RS_) values over time [21]. Two protective mechanical ventilation strategies, the TCAV method (**a**) and the ARDSNet (LVt) method (**b**), were tested in a clinically applicable 48-h porcine ARDS model of peritoneal sepsis (PS) and gut ischemia/reperfusion (I/R) injury [21]. The evolution of *C*_RS_, Δ*P*, and Vt with time in each group occurred over the 48-h study period (**c**, **d**). In the ARDSNet LVt method group, Δ*P* increased despite the reduction in Vt because of worsening *C*_RS_. With the TCAV method, Δ*P* remained low despite Vt = ~ 12 ml/kg because *C*_RS_ progressively increased (**c**, **d**). The personalized and adaptive Vt based on lung *C*_RS_ (i.e., high *C*_RS_ = large Vt and low *C*_rs_ = small Vt) was also seen in the brain-dead organ donor (Fig. 5a). Gross lung photos illustrate that the TCAV method (**a**) was lung protective, whereas the LVt method (**b**) resulted in severe acute lung injury. Δ*P* was calculated retrospectively and was not in the publication by Roy et al. [21]

With CPAP, the Vt is directly related to *C*_RS_ (Fig. 5a, top). The adaptive quality of the TCAV breath allows for unique personalization of Vt based on changes in lung physiology in contrast to the prevailing “one size fits all” 6 ml/kg method [83]. Further, the TCAV method maintains a low Δ*P* since Vt decreases as *C*_RS_ decreases (Figs. 5a, top and 7). Figure 7 presents gross lung photographs and the corresponding lung compliance (*C*_RS_), tidal volume (Vt), and driving pressure (Δ*P*) calculated from a previously published paper [82]. The animal model utilized was a clinically applicable porcine peritoneal sepsis and gut ischemia/reperfusion (PS + I/R) ARDS model [83]. Two groups of animals were studied: (1) ARDSNet low Vt (LVt) method applied after the animals desaturate and (2) the TCAV method applied immediately following PS + I/R injury. The time post-PS + I/R injury that these two protocols were applied matched the time of application on patients clinically (i.e., ARDSNet method is applied to patients after oxygen desaturation [2] and TCAV is applied immediately upon intubation [59]). In the ARDSNet group, *C*_RS_ continually decreased over the 48-h study period, whereas in the TCAV group, *C*_RS_ remained similar to baseline at T48 (Fig. 7c). The Δ*P* in the TCAV group remained in the normal range even with elevated Vt (12 ml/kg) because *C*_RS_ also increased (Fig. 7d). Gross photos indicate that the TCAV method (Fig. 7a) maintained an open homogeneously ventilated lung without edema, whereas the ARDSNet method (Fig. 7b) allowed the lung to develop severe atelectasis and both intra-lobule and airway pulmonary edema.

## Extended CPAP time and CO_2_ retention

Given that the inspiration:expiration (*I*:*E*) ratio for TCAV is approximately 12:1, CO_2_ retention could reasonably be a concern. Because the TCAV method is such an effective lung recruitment tool, there is seldom an issue with high blood levels of CO_2_ once the lung is fully recruited. Once recruited, there is a large surface area for CO_2_ diffusion and thus high concentrations of CO_2_ can be exhaled during the short release phase. The TCAV method can be applied preemptively as soon as the patient is intubated, never giving the lung a chance to collapse and eliminating any problems with CO_2_ retention [50], thus minimizing the risk of hypercapnia and eliminating the need for extracorporeal venovenous CO_2_ removal (ECCO_2_R). In addition, if the patient is adequately hydrated, there is no negative impact on lung perfusion since lung recruitment reestablishes normal FRC, which reduces pulmonary vascular resistance and right heart afterload [91, 92].

## Analysis of recent RCTs using the APRV mode

No human RCTs have yet utilized the TCAV method, but several recent RCTs have approximated many of the settings. Zhou et al. first evaluated 138 patients with a P/F less than 200 mmHg who were intubated for less than 48 h and randomized to receive either ARDSNet LVt or APRV with TCAV-like settings [93]. The APRV group demonstrated a significant decrease in number of days on mechanical ventilation (from 15 to 8), length of ICU stay (20–15), tracheostomy requirement (29.9% to 12.7%), and a 13.4% absolute decrease in mortality (34.3% to 19.7%, *p* = 0.053), although the study was not sufficiently powered to show a difference in mortality.

Ganesan et al. conducted an RCT using APRV and examined children under 12 years old with ARDS who had been intubated for less than 72 h and were randomized to receive either standard LVt strategy or APRV [94]. Unlike the Zhou trial, the APRV arm performed significantly worse, necessitating early trial termination. The investigators, however, introduced two significant and synergistically harmful changes to the TCAV protocol: setting and adjusting the *P*_High_ pressure of the CPAP phase based on Vt and improper regulation of spontaneous breathing.

By limiting *P*_High_ to maintain a lower Vt, the investigators never opened the lung to the point necessary to eliminate regional lung strain, the same mechanism hypothesized to explain the failed ART RCT. Their initial mean airway pressure (Pmaw) difference was only 1.6 cmH_2_O despite setting *P*_High_ at the Pplat and then adding an additional 2 cmH_2_O. The authors even provide a table for guiding initial *P*_High_ settings, which, based on the APRV arm’s P/F ratio of 124 mmHg, should have resulted in an initial Pmaw difference closer to 7 cmH_2_O—an almost 40% increase from what was observed.

Lastly, Hirshberg et al. conducted an RCT in adults with acute hypoxic respiratory failure and attempted to keep the Vt at about 6 ml/kg. The study was stopped early in part because the release volumes (i.e., Vt) often exceeded 12 ml/kg. Using the TCAV protocol an increasing Vt indicates that the lung is reopening and is associated with improved *C*rs, Δ*P*, and CXR (see example, Fig. 5b). In addition, there was no evidence that the Vt of 12 ml/kg caused VILI since there were no significant differences in PaO_2_/FiO_2_ (P/F) ratio, sedation, vasoactive medications, pneumothorax, or outcome between groups [95]. Lastly, the *T*_Low_ was not set to a strict *E*_FP_ × 75% = *E*_FT_.

The APRV mode using different application methods has recently been shown in statistical reviews and meta-analyses of RCTs to improved oxygenation, have a mortality benefit, and increase the number of ventilator-free days as compared to conventional ventilation strategies, without a higher risk of barotrauma or negative hemodynamic effects [96, 97].

## Conclusions

Neither the current lung protect and rest nor OLA ventilation strategies have been effective at reducing VILI and ARDS-related mortality below that in the ARMA study. For a protective ventilation strategy to be effective, it must open and stabilize the lung. Dynamic physiology of alveolar volume change suggests that the use of ventilation time can solve this heretofore intractable problem. The novel use of inspiratory and expiratory times to open and stabilize the acutely injured lung may accomplish the OLA goals where traditional ventilation strategies have failed. Specifically, the TCAV method, which uses an extended time at inspiration to open alveoli and brief expiratory time to prevent alveolar re-collapse has been shown to effectively open and stabilize the lung in animal ARDS models. There is a sound physiological rationale for the efficacy of the TCAV method, and deviations from this method may result in a significant loss of lung protection. The combination of basic science and clinical work has given this group a paradigm changing perspective. Our approach focuses on veiled mechanisms that have been largely overlooked, such as understanding the time necessary for the alveolus to open or collapse or taking advantage of biological realities, such viscoelasticity, to manage the lung. The new paradigm in TCAV is configuring each breath guided by the previous one, which achieves real-time titration of ventilator settings and minimizes instability induced tissue damage. This novel methodology changes the current approach to mechanical ventilation, from arbitrary to personalized and adaptive. The outcome of this approach is an open and stable lung, which reduces regional strain and provides greater lung protection.

## Data Availability

Not applicable.

## Abbreviations

ARDS: acute respiratory distress syndrome
VILI: ventilator-induced lung injury
APRV: airway pressure release ventilation
FRC: functional residual capacity
TCAV: time-controlled adaptive ventilation
CPAP: continuous positive airway pressure
TC-PEEP: time controlled-positive end expiratory pressure
*T*_Low_: time at low pressure
*T*_High_: time at high pressure
*P*_High_: pressure at inspiration
*P*_Low_: pressure at expiration
PEEP: positive end expiratory pressure
*E*_FT_: expiratory flow termination
*E*_FP_: expiratory flow peak
RCT: randomized controlled trial
OLA: open lung approach
MB_P_: mechanical breath pattern
CT: computerized axial tomography
